# Selective Extraction and Quantification of Hemoglobin Based on a Novel Molecularly Imprinted Nanopolymeric Structure of Poly(acrylamide-vinyl imidazole)

**DOI:** 10.1021/acsomega.4c00547

**Published:** 2024-04-15

**Authors:** Koray Şarkaya, Hilal Özçelik, Esra Yaşar, Timuçin Güner, Emre Dokuzparmak, Sara Hooshmand, Sinan Akgöl

**Affiliations:** † Department of Chemistry, Faculty of Science, Pamukkale University, Denizli 20160, Turkey; ‡ Department of Biochemistry, Faculty of Science, Ege University, Izmir 35100, Turkey; § Department of Bioengineering, Ege University, Izmir 35100, Turkey; ∥ Sabanci University Nanotechnology Research and Application Center (SUNUM), Tuzla, Istanbul 34956, Turkey

## Abstract

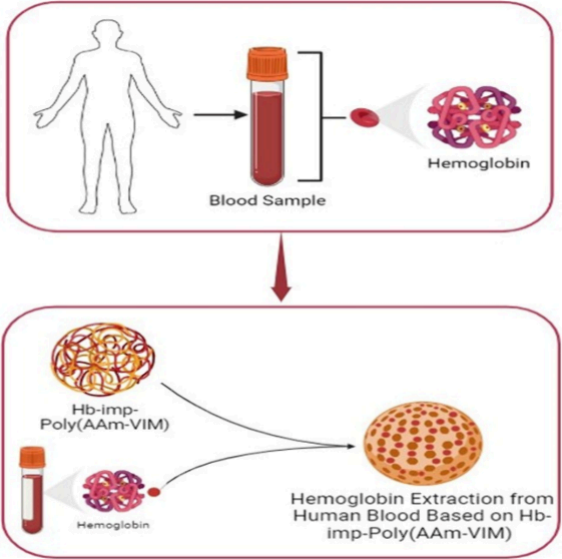

Imbalances in hemoglobin (Hb) levels can lead to conditions such as anemia or polycythemia, emphasizing the importance of precise Hb extraction from blood. To address this, a novel synthetic imprinted polymer was meticulously developed for capturing and separating Hb. Poly(acrylamide-vinylimidazole) nanopolymer (poly(AAm-VIM)) was synthesized using acrylamide and vinyl imidazole as functional monomers through surfactant-free emulsion polymerization. Characterization using FTIR, particle size, zeta potential, and SEM ensured the polymer’s structure. The Hb-imprinted nanopolymer (Hb-poly(AAm-VIM)) demonstrated notable specificity, with a calculated Hb-specific adsorption value (Q_max_) of 3.7377 mg/g in a medium containing 2.5 mg/mL Hb. The molecularly imprinted polymer (MIP) exhibited approximately 5 times higher Hb adsorption than the nonimprinted polymer (NIP). Under the same conditions, the imprinted nanopolymer displayed 2.39 and 2.17 times greater selectivity for Hb over competing proteins such as bovine serum albumin (BSA) and lysozyme (Lys), respectively. Also, SDS-PAGE analysis results confirmed the purification of Hb by the molecularly imprinted nanopolymer. These results underscore the heightened specificity and efficacy of the molecularly imprinted nanopolymer in selectively targeting Hb atoms among other proteins. Incorporating such polymers is justified by their notable affinity, cost-effectiveness, and facile production. This research contributes valuable insights into optimizing synthetic imprinted polymers for efficient Hb extraction, with potential in medical diagnostics and treatment applications.

## 1 Introduction

Hemoglobin (Hb) is a hydrophilic protein-rich structure originating in the bone marrow, stored within red blood cells, and crucial for oxygen transport from the lungs to body tissues. Its pivotal function is the transportation of oxygen from the lungs to body tissues, contributing to the characteristic coloration of red blood cells. Structurally, Hb takes the form of a tetramer, resulting from the amalgamation of four polypeptide chains—two pairs—each bearing an oxygen-binding heme group.^1^ In adult women, the reference range for Hb in blood typically falls between 12.2 and 15.5 g/dL, while in adult men, the range is generally 13.5 to 17.5 g/dL. These values serve as benchmarks to assess and evaluate the Hb levels in individuals, helping healthcare professionals in the diagnosis and monitoring of various health conditions.^2^ When Hb levels surpass the specified ranges, it signals potential health issues. Addressing Hb imbalances is crucial for managing and preventing associated health issues.^3^

As Hb stands as a crucial element in the blood, its analysis serves as a crucial diagnostic and therapeutic tool for numerous diseases. Various analytical methods, including chromatography,^4^ electrochemical sensors,^5^ surface plasmon resonance (SPR),^6^ and spectroscopic techniques,^7^ are at our disposal. However, each method comes with its distinct merits and drawbacks. Challenges such as elevated costs, prolonged analysis durations, and sustainability concerns, are prevalent. The aspiration is to devise an analytical approach that circumvents these limitations, offering cost-effectiveness, utility, and mitigation of associated disadvantages.^8^ The goal is to craft a method that is not only efficient but also addresses the drawbacks of existing techniques, ensuring practicality and affordability in its application.^9^ Fluorescence and electrochemical analyses, for instance, involve intricate and time-consuming processes utilizing blood samples, incorporating biochemical and physical steps for Hb detection through fluorescent and electrochemical signals, respectively.^10,11^ Spectrophotometry, in contrast, relies on optical direct detection, observing monochromatic light intensity changes caused by Hb or its derivatives’ absorption, following the Lambert–Beer law. Recent years have seen the emergence of optical-based detection methods, such as photoacoustic spectrometry, dynamic spectrometry, and spectral imaging, garnering significant interest from researchers. Photoacoustic spectrometry exploits the quantitative relationship between the power of the photoacoustic signal and Hb concentration, presenting ease of operation but faces challenges with weak signals easily overwhelmed by noise.^12^ Dynamic spectrometry relies on photoplethysmography signals in subcutaneous vessels, yet the complexity of skin’s optical properties can yield unstable results.^13^ Spectral imaging employs image analysis based on the spectral characteristics of the detection area but is hindered by the method’s complexity and stringent requirements for the detection area.^14,15^ While these methods offer valuable insights into the interaction between biology and light, facilitating noninvasive and real-time Hb detection, their sensitivity, accuracy, and stability are susceptible to interference during the detection process. Researchers continue to grapple with these challenges in pursuit of refining and enhancing the performance of Hb detection methodologies.^16^

Polymerization is a vital process in forming polymers where smaller molecules (monomers) combine chemically to create larger molecules or macromolecules. These macromolecules merge to generate the final polymers. Different methods are used, each tailored to produce polymers with distinct properties for specific applications. The chosen polymerization method significantly shapes the properties and potential uses of resulting polymers.^17^ The versatility of polymerization techniques enables customization of polymers to meet specific requirements in a wide range of industrial, medical, and technological applications. Polymers are broadly categorized into natural and synthetic types. Natural polymers, such as proteins, starch, cellulose, and DNA, constitute a significant portion of living tissues. In contrast, synthetic polymers exhibit a wide range of physical properties and are regarded as highly successful and valuable materials in various applications. The versatile nature of synthetic polymers has led to their widespread use across diverse fields.^18^ In contemporary applications, synthetic polymers play a pivotal role exemplifying the revolutionary impact of polymer advancements.^19^ Derived from natural sources, biopolymers are eco-friendly and sustainable, requiring fewer resources than petrochemical-based polymers.^20,21^ Widely used in industries such as food packaging, agriculture, and medicine, common biopolymers include cellulose, starch, proteins, and polyhydroxybutyrate (PHB), aligning with the increasing demand for sustainable alternatives.^22,23^

Molecularly imprinted polymers (MIPs) represent a well-established class of materials with a rich historical background and a diverse array of applications.^24,25^ In the realm of chemical analysis, MIPs have the potential to substitute biogenic materials like antibodies, providing the necessary selectivity for analytical methods.^26−28^ Beyond their various applications, the preparation of MIPs for analysis demands meticulous consideration of specific factors that play a critical role in influencing the overall efficiency of the imprinting process.^29^ MIPs exhibit distinctive selectivity for a predefined analyte or compounds sharing similar structures, featuring detection regions that prevent unintended binding.^30−32^ Their versatility finds application across various domains, including industry, medicine, agriculture, and the environment.^33^ The attractiveness of MIPs lies in their cost-effectiveness, simplicity, ease of production, physical durability, stability, resistance to temperature and pressure, applicability to numerous target analytes, and high affinity for these targets.^34^ The synthetic preparation of the polymer involves a multiple-stage process using the molecular imprinted polymer (MIP) method, a technique refined through specific steps including selection of target molecule and functional monomers, addition of initiators and cross-linkers, preparation of the molecular printing template, polymerization, template removal, cleaning, and characterization of the obtained polymer, and employing the obtained MIP for the recognition and extraction of the target molecule. Effective extraction techniques play a pivotal role in detecting biological analytes by facilitating the isolation and concentration of specific molecules from complex biological samples, thereby improving the precision and sensitivity of analytical methods.^35−39^ Applications include chemical separation,^40^ drug delivery,^41^ or biosensors.^29,42−44^

The detection of Hb from blood samples using molecularly imprinted polymers (MIPs) involves the creation of selective and specific binding sites within the polymer matrix that are designed to recognize and capture Hb molecules.^45−48^ MIPs can be intricately designed to distinctly identify and attach to Hb, thereby reducing interference from other constituents in the blood sample.^33,49,50^ MIPs demonstrate the advantageous quality of reusability, allowing them to be employed for numerous detection cycles.^51^ This characteristic contributes to cost-effectiveness and resource efficiency.^52^ MIPs commonly showcase robust stability, rendering them well-suited for practical applications. This inherent stability enhances the reliability and longevity of MIPs in the context of Hb detection, further solidifying their utility in various analytical settings.^9,53^

This research endeavors to synthesize a specialized polymer tailored for the specific capture of Hb from blood. The primary goal of this study was to develop a molecularly imprinted polymer designed for the highly selective extraction of Hb, employing acrylamide-vinylimidazole (AAm-VIM) as a main and functional monomer, separately. AAm was deliberately selected for its highly electrophilic reactivity and minimal toxicity, acting as the main monomer. Additionally, VIM was selected as a functional monomer to facilitate the formation of complexes with Hb, also incorporating its capacity to engage in intramolecular hydrogen bonding. The synthesis involved an optimized surfactant-free emulsion polymerization approach for producing both imprinted and NIP polymers. Furthermore, the investigation included the selective extraction of Hb from real blood samples with a focus on determining the optimal binding conditions. This approach offers a promising avenue for the development of selective and efficient methods for Hb detection in blood samples with potential applications in clinical diagnostics and research.

## 2 Results and Discussion

### 2.1 Characterization Studies

#### 2.1.1 FTIR Analysis

**Figure 1:**
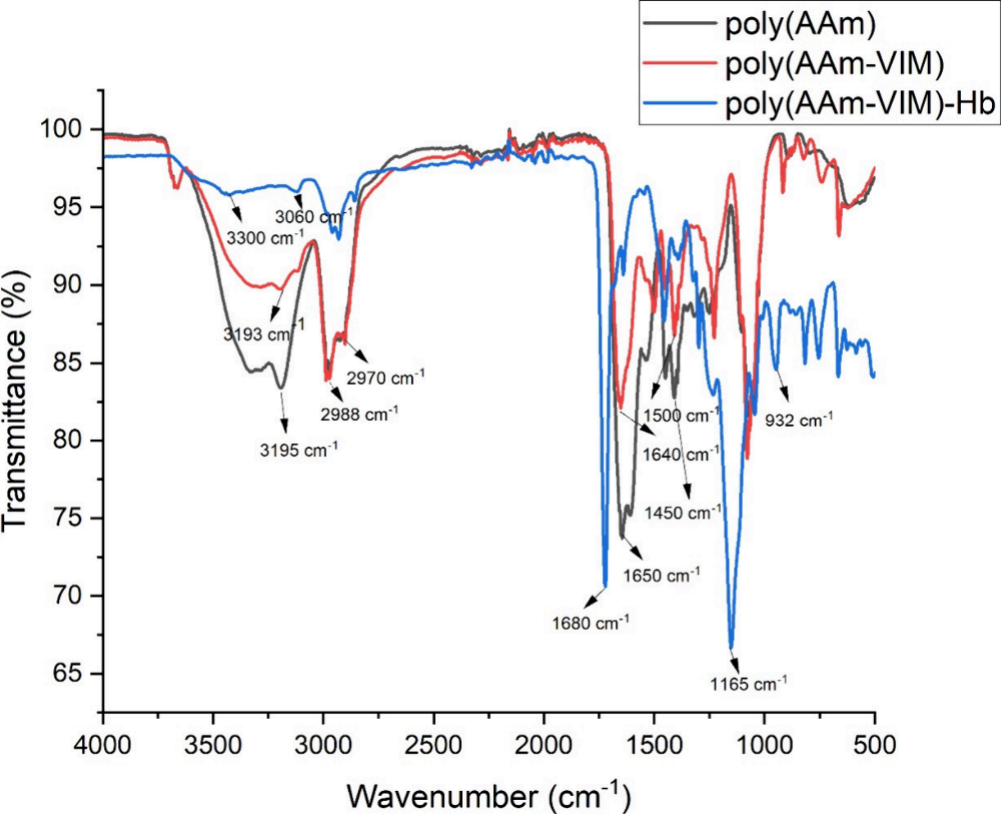
FTIR spectra of poly(AAm-VIM)-Hb, poly(AAm-VIM), and poly(AAm) nanopolymers.

FTIR spectra of synthesized MIP nanopolymer poly(AAm-VIM)-Hb, poly(AAm-VIM) and nonimprinted poly(AAm) nanopolymers for adsorption studies are presented in Figure 1. Accordingly, the absorption bands at 3430 and 3195.51 cm^–1^ for the poly(AAm-VIM) cryogel are defined as the stretching vibration of NH_2_. The absorption bands at 2988.20 cm^–1^ were interpreted as the C–H stretching of −CH and −CH_2_ in the main chain of the copolymer, and the absorption band at 1645 cm^–1^ corresponds to the C=O group of the AAm chain. The absorption bands at 1449 and 1500 cm^–1^ indicate the stretching vibrations of the C–C and N–C bonds in the VIM chain. The bands at 762 and 662 cm^–1^ signify the C–H ring bending vibration and C–N vibration of the azole ring, respectively, further confirming the presence of VIM in the structure. These characteristic AAm and VIM absorption bands confirm that the poly(AAm-VIM) nanopolymer was successfully synthesized. The presence of these characteristic absorption bands in the FTIR spectra confirms the successful synthesis of the poly(AAm-VIM) nanopolymer. The characteristic peaks of Hb for MIP nanopolymers are N–H stretching (3300 cm^–1^), N–H bending (3060 cm^–1^), C=O stretching and C–N stretching (1680 cm^–1^), and CO–O-C asymmetric stretching (1165 cm^–1^).^54^ These bands serve as fingerprints for the specific functional groups and chemical bonds present in the copolymer, providing evidence of its composition and structure.

#### 2.1.2 FESEM Analysis

**Figure 2:**
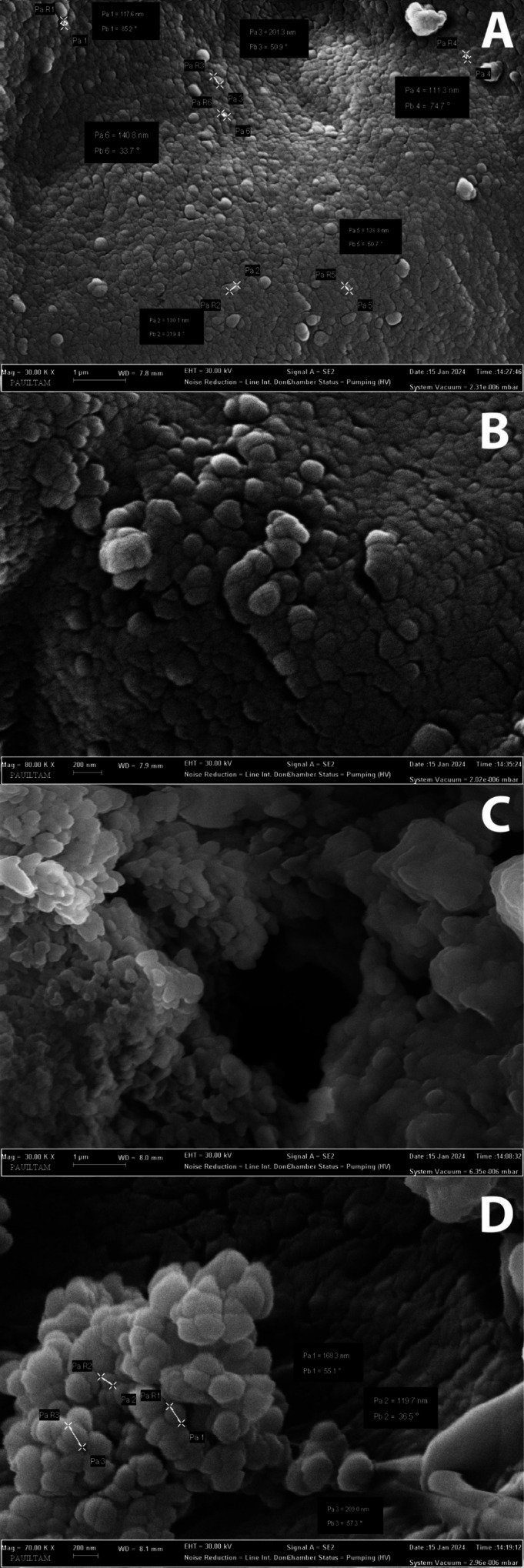
FESEM images of nanopolymers with different magnifications; (A, B) NIP nanopolymers; (C, D) MIP nanopolymers.

The scanning electron microscopy (SEM) images of both NIP and MIP nanopolymers reveal a distinct morphological structure (Figure 2A–D). The particles exhibit a spherical form, and their sizes appear to be consistent and compatible across different magnifications. The uniformity in size and the spherical structure observed in the SEM images provide valuable insights into the morphology and physical characteristics of the synthesized nanopolymer. This information is essential for understanding the structural features and potential applications of both NIP and MIP nanopolymers in various fields, such as drug delivery or biosensing.

#### 2.1.3 Zeta Potential and Size Analysis

**Figure 3:**
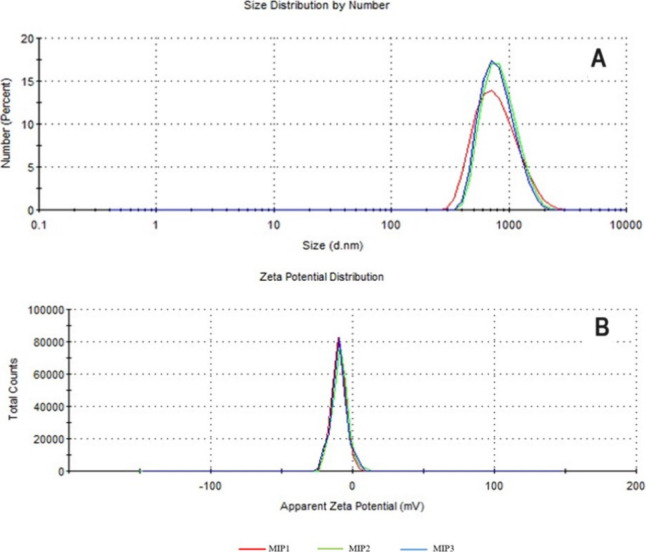
Zeta analysis for MIP nanopolymers: (A) size analysis and (B) potential analysis.

The average size of MIP nanopolymers (in 3 replicates of MIP1, 2 and 3) is 858 nm (Figure 3A). Additionally, MIP nanopolymers were measured as (−) 9.60 mV (Figure 3B). The dispersity of the nanopolymers was calculated as 0.102.

### 2.2 Optimization of Adsorption Conditions

#### 2.2.1 Hb Calibration Lines

The calibration lines were generated to establish a correlation between the absorbance values and concentrations of Hb solutions. These charts serve as a reference for accurately determining the concentration of Hb in subsequent experiments. The Hb calibration lines were generated through a meticulous process involving two calibration sessions. It is crucial to use consistent measurement conditions, including the same wavelength (406 nm) and spectrophotometer settings, for both Calibration I and Calibration II to ensure the accuracy and comparability of the results.

The details of each calibration session are as follows. First, aqueous solutions containing six different Hb concentrations, ranging from 0.1 to 2.5 mg/mL, were meticulously prepared. Utilizing a UV–vis spectrophotometer, the absorbance of each Hb solution was measured at a specific wavelength of 406 nm. A calibration line was constructed by plotting the absorbance values against the corresponding concentrations (mg/mL) of the Hb solutions (*y* = 0.1855*x* – 0.0035 (*R*^2^ = 0.9912). Following the pH screening, a new set of Hb solutions was prepared, this time in a pH 6.0 buffer solution. The absorbance of each Hb solution in the pH 6.0 buffer was measured using the UV–vis spectrophotometer at the established wavelength of 406 nm with the contribution of isosbestic point of Hb.^55^ A second calibration line was constructed by plotting the absorbance values against the corresponding concentrations (mg/mL) of the Hb solutions in the pH 6.0 buffer (*y* = 0.1571*x* + 0.0019 (*R*^2^ = 0.9987). These calibration lines serve as reference tools, establishing a quantitative relationship between absorbance values and the concentration of Hb solutions. They are vital for accurate Hb concentration measurements in subsequent experiments, providing a reliable basis for the spectrophotometric analysis.

#### 2.2.2 Investigating the Effect of pH on Hb Adsorption

To assess the impact of pH on Hb adsorption, buffers were prepared at 10 mM concentrations, each at varying pH levels—pH 5.0 (acetate), pH 6.0–8.0 (phosphate), and pH 9.0 (TRIS). The adsorption studies were conducted at room temperature with a final volume of 1.5 mL. A solution containing Hb and 10 mg of nanopolymer was introduced to an initial Hb concentration of 1.0 mg/mL, allowing for a 2 h adsorption period using the continuous adsorption method. The amount of Hb adsorbed by the polymer for each pH value was determined using the eq 1:
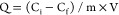
1

**Figure 4:**
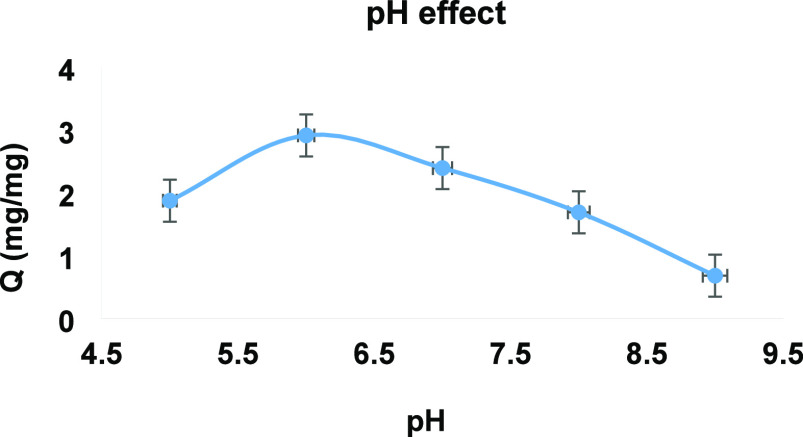
pH effect on Hb adsorption to an imprinted nanopolymer.

In this equation, Q represents the amount of Hb adsorbed per unit mass (mg/mg) of the nanopolymer, where C_i_ and C_f_ denote the Hb concentrations (mg/mL) in the initial solution and the aqueous phase after a specified time, respectively. Additionally, V stands for the volume of the aqueous phase (mL), and m (mg) represents the mass of the nanopolymer used. The resulting quantities were then graphed to identify the optimal pH conditions for effective adsorption (Figure 4).

The graph clearly illustrates a significant rise in the Hb adsorption capacity for the MIP nanopolymer as the pH shifted from 5.0 to 6.0. The peak Hb adsorption was identified at 2.93 mg/mg in pH 6.0 sodium phosphate buffer. Notably, Hb carries a neutral charge at pH 6.0, aligning with its p*K*_a_ value or isoelectric point.^56^ Theoretically, maximal adsorption is anticipated at the isoelectric point due to the nature of the adsorption process. The predominant forces governing the binding between the Hb molecule and the nanopolymer involve secondary interactions—such as hydrogen bonds, dipole–dipole interactions, electrostatic interactions, van der Waals interactions, and hydrophobic interactions. Among these, the hydrophobic interactions between the VIM (used as a functional monomer) and Hb, particularly within Hb-specific cavities, are presumed to be dominant.^57^

At pH 6.0, where the net charge is zero, heightened interaction occurs in the hydrophobic regions of the polymer, especially within the Hb-specific cavities. The hydrophobic character in these regions stems from the structure of the VIM comonomer. Contrastingly, at higher pH values, the Hb hydroxyl group carries a negative charge, leading to electrostatic repulsion in negatively charged regions on the polymer. This results in a reduction in the amount of adsorption.

Upon examination of the acidic region, it was observed that adsorption increased up to pH 6.0, experienced a slight decrease in the neutral region, and decreased again in the basic region. The fluctuation was hypothesized to be influenced by variations in ionic strength due to the different buffers used in the environments. Evaluating the relative change in Q values, it became apparent that adsorption in the sodium phosphate buffer at pH 6.0 was approximately 80% higher than that at other pH values. Consequently, a sodium phosphate buffer at pH 6.0 was identified as the optimum pH for Hb adsorption.

#### 2.2.3 Investigating the Effect of Temperature on Hb Adsorption

**Figure 5:**
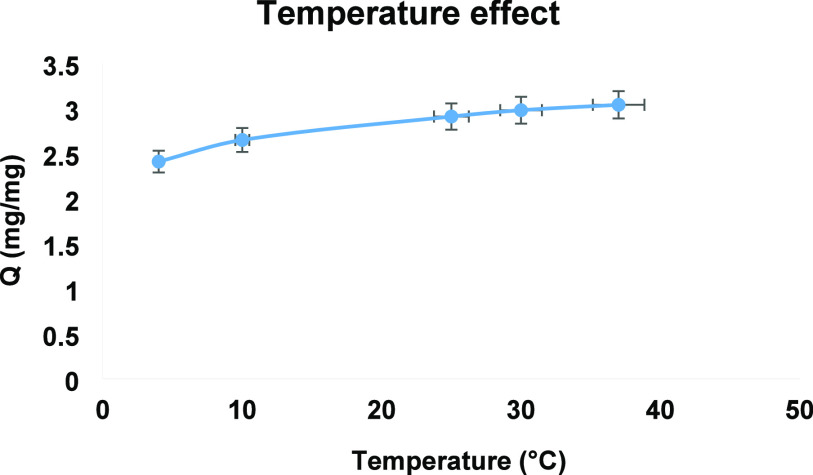
Temperature effect on Hb adsorption to an imprinted nanopolymer.

To investigate the impact of temperature on Hb adsorption, the study encompassed temperature values of 4, 17, 25, 30, and 37 °C. Conducted in a pH 6.0 sodium phosphate buffer, the experiments involved an initial Hb concentration of 1.0 mg/mL. 10 mg of polymer was added to a 1.5 mL volume, and the mixture was stirred for 1 h under conditions adjusted to the adsorption temperature. Following adsorption, the samples underwent centrifugation at 14,100*g* for 20 min, and Hb analysis was conducted on the resulting supernatants. To ascertain the initial concentration, control experiments devoid of a polymer were conducted. These blind experiments involved mixing in a water bath for 2 h at each temperature value, serving as a baseline for comparison. Figure 5 illustrates the variation in the adsorption capacity of the Hb-imprinted nanopolymer with the temperature. The investigation delved into the temperature range of 4–37 °C. The graph reveals an escalating trend in the Hb adsorption capacity of the MIP nanopolymer with rising temperature values. The binding capacity demonstrated a consistent increase, reaching its pinnacle at 37 °C, indicating that higher temperatures enhance the binding efficacy.

The dominance of hydrophobic interactions in adsorption was attributed to both the VIM monomer situated in Hb-specific cavities on the nanopolymer surface and the hydrophobic nature of the target molecule Hb. In instances where both interacting elements are hydrophobic, as observed in this case, hydrophobic interactions tend to prevail in the adsorption process.

The process of binding hydrophobic substances in water adheres to the established theorem: G = (H – TΔS), where G represents the free energy change, H is the enthalpy change, T signifies temperature, and ΔS denotes entropy. In this scenario, with hydrophobic adsorbents, the driving force is entropy, leading to an increase in the interaction with temperature. Notably, H can be either positive or negative, but the positive entropy change effectively controls G. Consequently, as the temperature rises, entropy experiences an increment.

Additionally, weak interactions, such as hydrogen bonds and van der Waals interactions, were observed with the hydroxyl groups on the AAm surface. This multifaceted interaction profile, incorporating hydrophobic interactions and other weak forces, contributes to the overall binding affinity and underscores the temperature-dependent nature of the adsorption process.

#### 2.2.4 Effect of Initial Concentration on Hb Adsorption

To explore the influence of initial concentration on Hb adsorption, the adsorption medium was set with initial Hb concentrations ranging from 0.1 to 2.5 mg/mL. The final volume was standardized at 1.5 mL, achieved by adding 3 mg/mL Hb stock to pH 6.0 sodium phosphate buffer to attain the desired concentration. Precise pipetting procedures were employed followed by the addition of 10 mg of polymer. The process was conducted at room temperature. Subsequent to the adsorption phase, the samples underwent centrifugation at 14,100*g* for 20 min, and Hb analysis was carried out on the resulting supernatants. To establish baseline values for each concentration level, blind trials devoid of a polymer were conducted. These trials involved the same procedure, and the outcomes were used to determine the initial concentration for each concentration value in the experimental setup.

**Figure 6:**
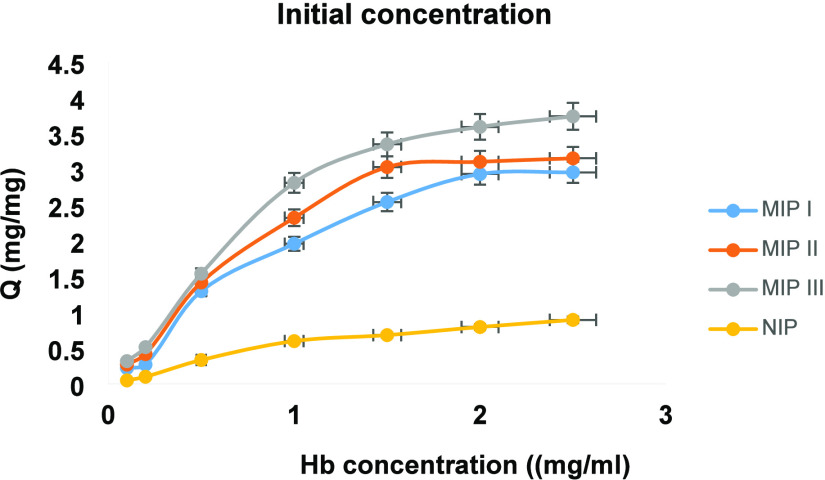
Effect of initial concentration on Hb adsorption (pH 6.0 sodium phosphate buffer, at 37 °C, adsorption: 1 h MIP (I,II and III)).

The impact of the initial Hb concentration on Hb adsorption is elucidated in Figure 6. The graph reveals a noteworthy observation: MIP III exhibited a higher binding capacity for Hb compared to other MIPs. This phenomenon is attributed to the utilization of a greater number of functional monomers, resulting in the creation of more binding sites. Concurrently, the graph illustrates that as the concentration of Hb in the solution increased, the amount of Hb adsorbed by nanopolymer per unit mass also increased, eventually reaching equilibrium at a value approximately four times higher than the polymer amount.

The correlation between the Hb concentration and adsorption amount can be explained by the rise in the concentration difference (C), which serves as the driving force for adsorption. As the driving force intensifies, the adsorption capacity increases, aligning with expectations. Notably, when the Hb concentration reached 2.5 mg/mL, the cavities on the molecularly imprinted nanopolymer designed for Hb were saturated, reaching the maximum adsorption capacity under the given environmental conditions.

The exceptional ability of the Hb-imprinted nanopolymer (Hb-poly(AAm-VIM)) to adsorb substantial amounts of Hb per unit mass is attributed to the extensive surface area of the nanostructure. Furthermore, the hydrophobic nature of VIM, positioned in molecular cavities specific to Hb, enhances the affinity for Hb, contributing to the impressive adsorption performance of the nanopolymer.

#### 2.2.5 Effect of Ionic Strength on Hb Adsorption

**Figure 7:**
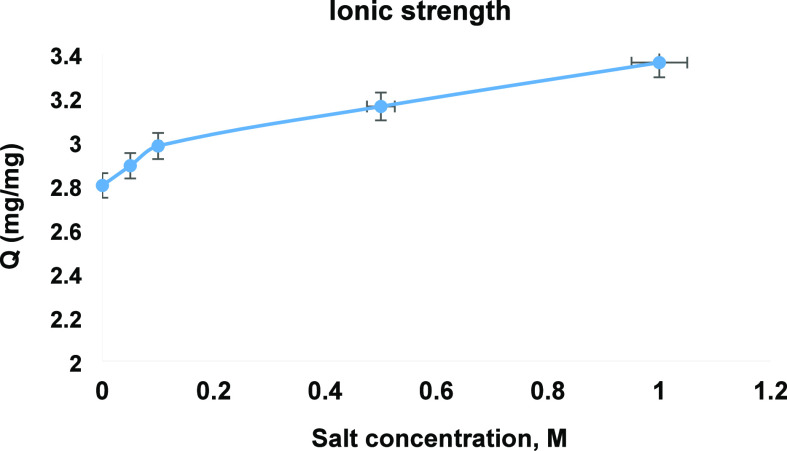
Effect of the ionic strength on Hb adsorption to imprinted nanopolymer.

To assess the impact of ionic strength on Hb adsorption, phosphate buffers with a pH of 6.0 and a concentration of 20 mM were prepared, incorporating varying NaCl concentrations ranging from 0 to 1.0 M. The experimental samples were subjected to an angled mixer set at 30 rpm and maintained at room temperature. The adsorption process onto the MIP nanopolymer was allowed to proceed for 1 h, after which the Hb content was quantified using a UV–vis spectrophotometer. This methodology enables the exploration of how different ionic strength conditions, modulated by NaCl concentrations, influence the adsorption efficiency of Hb onto the molecularly imprinted nanopolymer (Figure 7).

The observed trend indicates that the influence of ionic strength on protein adsorption becomes more pronounced with increasing salt concentration. At elevated salt concentrations, protein adsorption is heightened, primarily due to the diminished lateral electrostatic repulsion between adsorbed protein molecules. This reduction in repulsion leads to an increased protein density at the surface.

Furthermore, the impact of multivalent ions in the solution proved to be particularly significant. These ions have the capacity to bind to charged regions of the protein, neutralizing the charge density. This, in turn, increases the hydrophobicity of the protein, creating a notable preference for adsorption on the surface. It is crucial to note that the effect of the ionic strength is contingent on whether the protein charge aligns with or opposes the surface charge. The modulation of this interplay influences the overall adsorption behavior. The observed phenomena highlight the intricate interplay of electrostatic forces, charge neutralization, and hydrophobic interactions in the context of protein adsorption under varying ionic strength conditions.

### 2.3 Specificity and Selectivity Studies

Following the optimization of adsorption studies in aqueous solutions, the selectivity performance of the Hb-imprinted poly(AAm-VIM) nanopolymer was scrutinized. Bovine serum albumin (BSA) and lysozyme (Lys) were chosen as competitive proteins for this evaluation. Aqueous solutions containing the selected proteins at concentrations of 1.0 mg/mL were individually exposed to Hb-imprinted and NIP nanopolymers for a duration of 1 h.

The amounts of adsorbed amino proteins were determined at a wavelength of 595 nm by using the Bradford method. The selectivity coefficient (K_d_) of Hb, Lys, and BSA was subsequently calculated using eq 2.
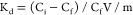
2

In eq 2, the selectivity coefficient (k) is determined by taking the ratio of the selectivity coefficient of the template molecule (Hb), denoted as K_d_^1^, to the selectivity coefficient of the competing proteins, denoted as K_d_^2^. This ratio provides a measure of the relative binding affinity of Hb compared to those of the competing proteins.

The selectivity coefficient (k) for binding Hb in the presence of competitive proteins was determined according to the following equation (eq 3):
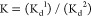
3

In eq 3, the relative selectivity coefficient (k′) is calculated as the ratio of the selectivity coefficient in the suppressed state (k_imprinted_) to the selectivity coefficient in the nonimprinted state (k_nonimprinted_). This ratio offers insight into how molecular imprinting affects the selectivity of protein binding considering the presence and absence of competition from other proteins.

The relative selectivity coefficient (k’), which gives an idea about the effect of imprinting on protein binding selectivity, is defined by the following equation (eq 4):
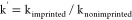
4

This methodology allows for the assessment of the Hb-imprinted poly(AAm-VIM) nanopolymer’s selectivity in binding specific amino acids over others in the presence of competitive proteins, shedding light on the molecular recognition capabilities of the imprinted nanopolymer.

The comparison of adsorption capacities reveals a notable distinction, with the molecularly imprinted nanopolymer exhibiting approximately 5 times more Hb adsorption compared to the NIP. Despite the similarity in particle diameters and specific surface areas between NIP and MIP, the significant disparity in adsorption capacity underscores the substantial impact of the imprinting process on adsorption. The incorporation of hydrophobic cavities specifically designed for the target molecule, Hb, on the polymer surface contributes to the observed increase in the adsorption capacity. Table 1 further illustrates that under the same environmental conditions the Hb adsorption of the MIP nanopolymer is 2.39 and 2.17 times more selective than the competing proteins BSA and Lys, respectively.

**Table 1:** K_d_, k, and k’ Values for BSA and Lys Relative to Hb

	**MIP**		**NIP**		
**protein**	K_d_ (mL/g)	k	K_d_ (mL/g)	k	k′
Hb	0.177		0.038		
BSA	0.074	2.39	0.025	1.52	1.57
Lys	0.08	2.17	0.034	1.12	1.94

This selectivity is particularly noteworthy, given the similar molecular structures of the proteins involved. The heightened adsorption of Hb compared to other proteins indicates that the polymeric structure has acquired selective character through the specific imprinting process. The polymer exhibits a significantly greater affinity for the target molecule, emphasizing the success of the molecular imprinting technique in conferring selectivity to the adsorption process.

### 2.4 Desorption and Reusability

**Figure 8:**
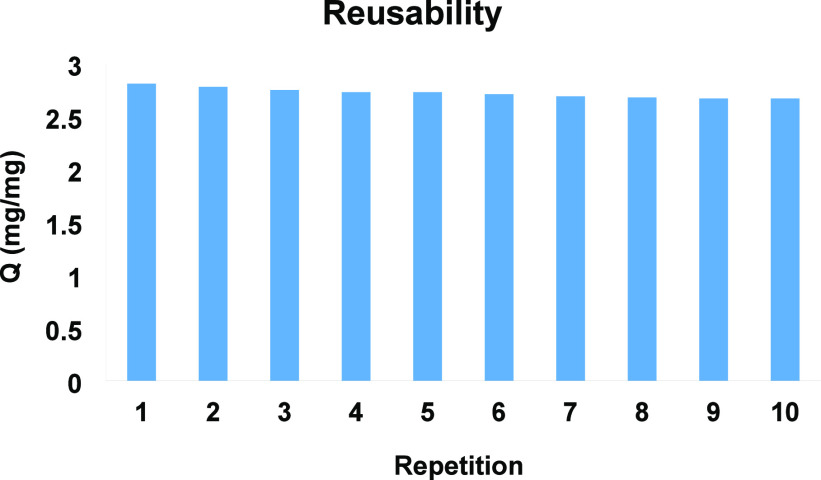
Adsorption–desorption cycle for Hb by the Hb-poly(AAm-VIM) (MIP III) nanopolymer. Desorption conditions: initial Hb concentration: 1.0 mg/L; volume: 1.5 mL, T: 25 °C, desorption time: 1.0 h, weight of dry nanopolymer: 10 mg.

The adsorption–desorption cycle showing the reusability of Hb-imprinted-poly(AAm-VIM) (MIP III) is shown in Figure 8. In the adsorption study of nanopolymers repeated at least 10 times, the desorption rate did not fall below 95%, respectively. Despite this desorption rate, which can be considered to be high, no significant decrease in Hb adsorption was observed. As a result of the large surface areas inherent in nanopolymers, the adsorption–desorption cycle occurred quickly. Accordingly, it was observed that adsorption–desorption studies reached equilibrium in less than 30 min.

### 2.5 SDS-PAGE Analysis

**Figure 9:**
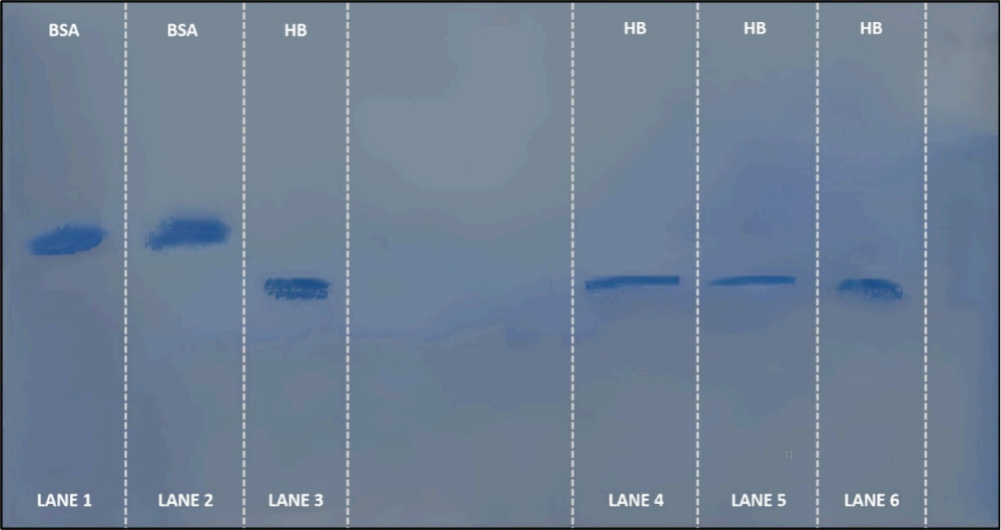
SDS-PAGE image: Lanes 1 and 2, biomarker (BSA); Lane 3, initial solution before Hb purification (1.0 mg/mL); Lanes 4 and 5, solution after Hb purification; Lane 6, desorbed a Hb solution.

The sodium dodecyl sulfate polyacrylamide gel electrophoresis (SDS-PAGE) method is frequently used to determine the molecular weights of proteins and the purity levels of purified proteins. In this method, proteins are run on a gel in the presence of SDS. It is possible to distinguish each protein and its subunits by forming a single band on the gel. SO_3_H, the active group of the Coomassie Brilliant Blue G-250 dye used in the study, binds to the free amino group of proteins in an acidic environment. Excess dye in the gel was removed by washing it five times with a solution containing 40% methanol (v/v), 10% acetic acid (v/v), and 50% water (v/v), and the solution was changed every 20 min. Bovine serum albumin (BSA) was used as the protein standard in the electrophoresis application. The molecular weight and purity of Hb purified from aqueous solution with poly(AAm-VIM) nanoparticles were checked by SDS-PAGE. As seen in Figure 9, the intensities of the bands belonging to Hb molecules decrease significantly after the adsorption process (64 kDa, Lane 3). Purified Hb is seen in rows 4–5–6. The purity of purified Hb was found to be 95.5%. In lanes 1 and 2, BSA protein is included as the reference molecule.

## 3 Conclusions

This study presents the synthesis of a novel molecularly imprinted nanosized hydrophobic support material, eliminating the need for an activation process in Hb removal. A system employing the Hb molecular imprinting technique (MIP) has been developed, offering a direct and efficient means of Hb removal. The resulting Hb-poly(AAm-VIM) nanopolymer is characterized as a stable, cost-effective, and easily prepared product with molecular memory. The application scope of the Hb-poly(AAm-VIM) (MIP III) nanopolymer extends to the separation and purification of Hb, making it particularly valuable for diagnostic purposes in biosensor applications. The system addresses the need for the rapid and effective removal of Hb, achieving this in a shorter time and at a lower cost while utilizing minimal support material.

This nanobiotechnological product is positioned as a high-tech solution, promising substantial benefits in terms of process innovation and efficiency. Its utility spans both natural and artificial plasmas, showcasing its versatility and potential for widespread applications. Furthermore, the study is positioned as an original contribution to the field of molecular imprinting, as evidenced by its potential impact on the international literature and patent potential. The innovative approach to Hb extraction and purification sets the groundwork for advancements in various domains, making it a noteworthy and impactful endeavor.^58^

## 4 Experimental Section

### 4.1 Materials and Apparatus

Acrylamide (AAm), *N*-vinyl imidazole (VIM), poly(vinyl alcohol) (PVA), sodium dodecyl sulfate (SDS), sodium bicarbonate (NaHCO_3_), ethylene glycol dimethacrylate (EGDMA), ammonium persulfate (APS), and sodium bisulfite (NaHSO_3_) was purchased from Sigma-Aldrich. All chemicals used in this context are of analytical purity, underscoring the precision and reliability of the materials in the experimental processes.

In the experimental procedures, the laboratory facilities of Ege University-Department of Biochemistry (BIOREGE) were utilized for various devices and experiments. The synthesis of polymers involved the use of a hot water bath (Wisd Wise Bath WSB-30). For washing and settling processes, a centrifuge was employed, with specific models including Centrion Scientific Benchtop centrifuges K2015R (UK) and Beckman Coulter Avanti centrifuge J-E, as well as Eppendorf minispin+. Adsorption experiments were conducted using a rotator (Wisd WiseMix RT-10). UV–vis spectra were obtained using a spectrophotometer (ThermoScientific Evolution 60). The precision balance used was a KERN&Sohn GmbH ABS220–4 model. The pH meter utilized was İSTEK NeoMet and an oven (Memmert, UNB400) was also applied. For homogeneously mixing solutions, a magnetic stirrer (Dragon lab, MX-F) was employed. The synthesized polymers’ zeta potential and size analysis were examined with Nano Zetasizer (NanoS, Malzern Instruments, London, England) at Ege University Nuclear Sciences Institute Laboratories.

### 4.2 Preparation of Hb imprinted nanopolymer (Hb-poly(AAm-VIM))

In the conducted studies, three distinct precomplexes were formulated to determine the optimal conditions. To achieve this, three different complexes were created and tested, each comprising 10 mg of Hb with varying volumes: 90.5 μL (MIP I), 181 μL (MIP II), and 271.5 μL (MIP III), separately. The synthesis process involved the following steps:

1. First Water Phase: 50 mg of PVA and 50 mg of SDS were added to 100 mL of DI H_2_O and mixed at 60 °C for 30 min.
2. Second Water Phase: 93.5 mg of PVA and 14 mg of SDS were dissolved in 10 mL of DI H_2_O at 60 °C. Then, 12.5 mg of sodium bicarbonate (NaHCO_3_) was added to the mixture.
3. Preparation of Hb-Functional Monomer Precomplex: A mixture containing three different ratios of Hb (template molecule) and VIM (functional monomer) was prepared. This mixture was dissolved in Eppendorf tubes. These mixtures were mixed with a rotator for 3 h to preorganize the Hb and VIM complex to obtain the precomplex.
4. Organic Phase: 70 mg of AAm with 1 mL of EGDMA was mixed with the precomplex.
5. Polymerization: The Second Water Phase and the Organic Phase were mixed at 400 rpm at 37 °C. Then, the First Water Phase was added to the mixture. The resulting mixture was exposed to nitrogen gas for 15 min. Subsequently, 100 mg of APS and 50 mg of NaHSO_3_ were added to the mixture, maintaining a flow of nitrogen gas. This mixture was stirred in a hot water bath at 100 rpm at 37 °C for 24 h.
6. Centrifugation: After polymer formation was observed, the polymers were centrifuged at 4000 rpm.
7. Purification: The polymers were dissolved in a 50:50 H_2_O:ethanol mixture to eliminate impurities. Then, the mixture was centrifuged for 10 min to precipitate the polymers.
8. Drying: The centrifuged polymers were dried in an oven at 30 °C for 24 h.

NIP was also prepared by using the same method, excluding the template molecule (Hb) from the polymerization medium. This comprehensive process outlines the steps involved in the synthesis, purification, and drying of molecularly imprinted polymers.

Following the synthesis of Hb-imprinted nanopolymer, a thorough washing procedure was implemented to eliminate any residual unreacted initiators and monomers. This involved multiple washes with 0.1 M NaCl. For each washing step, the solution was subjected to centrifugation at 14100 g for 20 min, resulting in the separation of the nanopolymer from the washing medium. After the washing process was completed, a desorption agent, 0.5 M NaCl, was employed to remove the template molecule, Hb, from the polymeric structure. The desorption process was conducted at 12-h intervals, precipitating the polymer and transferring it to fresh desorption solution. This cyclic process continued until Hb could no longer be detected in the desorption solution. Subsequently, the nanopolymer, now devoid of the template Hb molecule, was redispersed in 70 mL of DI H_2_O. The reconstituted nanopolymer was stored at 4 °C, ensuring their stability until further use. This meticulous series of washing and desorption steps ensured the purification and successful removal of the template molecule from the synthesized Hb-imprinted nanopolymer.

### 4.3 Characterization Studies

FTIR spectra of monomers were acquired by using an FTIR spectrophotometer (FTIR Prestige 21, Shimadzu). The surface morphology of the nanopolymers was thoroughly examined using field emission scanning electron microscopy (FESEM). Dried nanopolymer samples were covered with a gold–palladium combination to achieve this goal. The surface morphological features of polymer samples were investigated through studies conducted with a Zeiss Supra 40VP SEM. Zeta potential and size analysis of the synthesized nanopolymers were also examined. For this purpose, surface charge measurement was performed by injecting 1 mL of polymer solution into the sample chamber of the zeta dimension analysis device.

### 4.4 Optimization of Adsorption Conditions of Hb

By systematically examining these factors and analyzing the resulting data, the optimal conditions for Hb adsorption on a Hb-poly(AAm-VIM) nanopolymer can be determined. In the optimization experiments for Hb adsorption conditions of the Hb-imprinted nanopolymer, the effects of pH, temperature, ionic strength, and initial Hb concentration were systematically investigated. The following details the experimental setup: Hb solution at a concentration of 1 mg/mL was prepared in pH 6.0 phosphate buffer to be used in adsorption experiments. For pH experiments, buffers were prepared at pH values of 5.0, 6.0, 7.0, 8.0, 9.0, and 10 mM. Adsorption capacity value (Q) was measured with UV–vis spectrophotometry analysis, and the amount of Hb (mg) adsorbed per mg polymer was calculated.

### 4.5 Desorption and Reusability

0.5 M NaCl solution was used as a desorption agent for the desorption of Hb adsorbed by nanopolymers. Before starting the desorption process, it was centrifuged at 15,000 rpm for 2 h and precipitated to remove impurities and other unbound residues that the nanopolymers may contain. Under these conditions, nanopolymers were desorbed with the desorption solution on a rotator at room temperature for 2 h. After desorption, the nanopolymers were washed with deionized water and centrifuged again for reuse. Subsequently, the adsorption–desorption process was repeated ten times by centrifuging and using the same nanopolymers to determine the reusability of nanopolymers. The desorption rate of nanopolymers was calculated via eq 5.
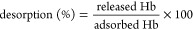
5

### 4.6 Sodium Dodecyl Sulfate Polyacrylamide Gel Electrophoresis (SDS-PAGE)

Hb purified from an aqueous solution, SDS-PAGE, was used to check the purity of the desorbed Hb. The most appropriate SDS-PAGE corresponding to the molecular mass of Hb (64 kDa) was performed with a 10% acrylamide (w/v) separation gel (1.0 mm). Preadsorption, postadsorption, and desorption samples of Hb solution were loaded onto the gel by mixing the sample with buffer containing 20% glycerol (v/v), 20% mercaptoethanol (v/v), 4% sodium dodecyl sulfate (SDS) (w/v), and 0.02% bromophenol blue (BPB) (w/v) at the same rate. The gel was mixed with a sample loading buffer, and protein concentrations were adjusted to 15 μg/mL for each sample. The gel was allowed to run for 5 h at 120 V with the loaded samples and in the presence of running buffer (Tris-glycine, pH 8.8) containing 0.1% SDS. After the application process, it was transferred to a Coomassie Blue G-250 dye and kept in the solution overnight.
